# Broadening the scope of social support, coping skills and resilience among caretakers of children with disabilities in Uganda: a sequential explanatory mixed-methods study

**DOI:** 10.1186/s12889-022-13018-x

**Published:** 2022-04-08

**Authors:** Mariam Namasaba, Neo Kazembe, Georgina Seera, Ali Ayub Baguwemu

**Affiliations:** 1 Department of Community and Global Health, Graduate School of Medicine, The University of Tokyo, 7 Chome-3-1 Hongo, 113-8654 Bunkyo City, Tokyo, Japan; 2 Center for African Area Studies (CAAS), Kyoto University, 46 Shimoadachi-cho Yoshida, Sakyo-ku, 606-8501 Kyoto, Japan; 3 Department of Psychology, Kyambogo University, P.O Box 1, Kampala, Uganda

**Keywords:** Children with disabilities, Coping skills, Social support, Resilience

## Abstract

**Background:**

Most caretakers of children with disabilities (CWDs) have adverse health outcomes. Approximately 31% of the caretakers have clinical depression in the world. In Sub-Saharan Africa, 42% of them face severe psychological distress. Caretakers in Africa face additional cultural challenges that undermine their coping skills, access to social support, and resilience.

**Methods:**

This study used sequential explanatory mixed methods to examine the relationships of social support, coping skills and resilience among caretakers of CWDs in Uganda. A total of 621 caretakers were surveyed, and 43 of them participated in interviews. Hierarchical cluster analysis and binary logistic regression were conducted to determine coping patterns and predict caretakers’ likelihood of using them. Hierarchical linear regression and thematic analyses then explored the relationships and perceptions of coping skills and resilience related to social support. A joint display was used to integrate results and show the convergence and expansion of quantitative and qualitative results.

**Results:**

Quantitative and qualitative findings converged that caretakers who received social support used adaptive coping skills and had higher resilience. Qualitative results expanded the finding that caretakers who received formal social support perceived it as a safer mode of care than informal social support.

**Conclusions:**

The study expanded the scope of social support, coping skills, and resilience. Caretakers perceived formal social support from schools as a safe mode of care that enabled them to use adaptive coping skills and have high resilience. Therefore, enrolling children with disabilities in schools at an early age is beneficial for building the resilience of their caretakers.

**Supplementary Information:**

The online version contains supplementary material available at 10.1186/s12889-022-13018-x.

## Background

Disability is the experience of having ineffective participation in society due to a long-term physical, intellectual, mental, or sensory limitation that interacts with an external barrier that an individual faces [1]. In children, disability includes congenital abnormalities, developmental impairments, or special educational needs [2, 3]. A total of 93 million children have mild or moderate disabilities globally [4, 5]. Of them, 13 million have severe impairments and greatly rely on their caretakers for sustenance [5].

Most caretakers of children with disabilities (CWDs) have negative caring experiences and adverse health outcomes [6, 7]. Globally, 31% of caretakers of CWDs (now referred to as caretakers) experience clinical depression [8]. In low- and middle-income countries, approximately 58% of the caretakers face psychological distress [9, 10]. In Sub-Saharan Africa, 42% of them face severe psychological distress compared to 36% of caretakers in high-income countries [11, 12]. The caretakers in Africa face additional cultural and educational constraints that affect their psychological wellbeing [13].

In Uganda, many communities believe that caretakers who committed past sins produce CWDs as payment for their shortcomings [14]. Some communities also consider CWDs as “not fully human” but mere habitat for evil spirits [15]. As a result, CWDs are often mocked by their peers and receive impartial attention from school staff compared to their non-disabled peers [16]. To promote the wellbeing of people with disabilities, the Ugandan government conducts community-based rehabilitation programs across 51 of its 135 districts [17]. A previous qualitative study conducted among caretakers in Uganda reported that those who receive formal social support through community-based rehabilitation gain practical coping skills and have positive caring experiences [18]. However, few CWDs (35%) can access formal social support through community-based rehabilitation  programs.

Social support is necessary for caretakers to have positive caring experiences [19, 20]. Social support is the emotional or instrumental assistance given to an individual or their beneficiary [21]. It can be conceptualized in relation to its components or source [22]. Regarding its components, social support is a coping skill whereby an individual seeks or receives emotional and instrumental support through different networks [17, 23]. Regarding its source, social support is a resource accessed through formal or informal sources [24]. Family or friends provide informal social support, and formal social support is provided by professionals such as medical personnel or school staff [25–27]. Caretakers who receive social support feel cared for and well-equipped to provide adequate care for their children [27, 28]. Social support is especially crucial in low-income settings where caretakers face many challenges that undermine their coping skills and resilience [29].

Coping skills and resilience are closely related [30]. Coping skills are psychological or physical responses that individuals use to overcome difficult situations [31]. Two widely used dimensions of coping skills are adaptive and maladaptive coping skills [32, 33]. Adaptive coping skills include social support (emotional and instrumental), acceptance, positive reframing, and active coping [34]. Maladaptive coping skills include substance use, behavioral disengagement, and denial [35]. Caretakers who use adaptive coping skills tend to have higher resilience than those who use maladaptive coping skills [36].

Resilience is the ability of an individual to overcome a challenging situation and maintain good health outcomes [37]. Resilience can be enhanced through access to protective factors such as social support and adaptive coping skills [38]. Individuals with high resilience often have better psychological and physical health outcomes than those with low resilience [39]. However, the relationships of social support, coping skills, and resilience vary across contexts and study methodology.

For instance, a quantitative study conducted in Ghana used a multidimensional scale to examine the association between social support and resilience. The study found that caretakers of CWDs who received informal social support had higher resilience than those who received formal social support [40]. Another quantitative study which used the same multidimensional scale to examine the association between social support and parental stress among caretakers of CWDs in Brazil, found no association between informal social support and perceived stress [41]. On the other hand, a qualitative study was conducted among caretakers of children with autism in Australia, and it found that caretakers increasingly relied on formal social over a period of nine years [42]. However, in two qualitative studies conducted in Uganda, caretakers who received informal social support had more time to participate in social activities with family and friends [43, 44].

While previous studies provide important insights into the relationships of social support, coping skills, and resilience among caretakers, they demonstrate variable findings on the relationships of social support, coping skills, and resilience, depending on the context of the caretakers. Furthermore, in Uganda, so far, the two previous qualitative studies we found contradicted each other with one highlighting formal social support and the other informal social support as beneficial for improving caretakers’ coping skills but not their resilience [18, 43]. Since mixed methods can illuminate the underlying aspects of social support that influence differences in caretakers’ coping skills and resilience [45]. This study adopted a mixed methods design and had two objectives:

1. To examine the relationships of social support, coping skills, and resilience among caretakers of children with disabilities in Uganda.
2. To explore the perceptions of coping and resilience among caretakers of children with disabilities in Uganda.

## Methods

### Study design and site

This was a sequential explanatory mixed-methods study [46]. The study design was selected because it was the best approach for understanding the multiple facets of social support, coping skills, and resilience among caretakers of CWDs [47]. The study was conducted in inclusive and special needs schools in the Kampala Capital City Authority, Uganda (KCCA). There were 15 schools for CWDs, under KCCA [48]. Of the 15 schools, seven were special needs schools (admitted only children with disabilities), and eight were inclusive schools (admitted both children with and children without disabilities). All the 15 schools were invited to participate, and data were collected between May and August 2018.

### Synchronization of mixed methods

The sequence and dependence of the quantitative and qualitative phases were based on the guidelines proposed by two previous mixed methods studies [47, 49].

### Implementation

The quantitative data were collected and analyzed from May through July 2018. The quantitative phase aimed to examine the first objective of the study. Qualitative data were then collected in August 2018, following preliminary quantitative data analyses. The qualitative phase was designed to examine the study’s second objective. Qualitative data were also used to explain the association between coping skills and resilience among caretakers. At the beginning of the study, quantitative and qualitative phases were connected through the formulation of open-ended interviews [47].

### Integration

The study integrated quantitative and qualitative results at three points using a dialogic process [50]: the intermediate stage (transition from quantitative to qualitative phase), the analysis of results, and the interpretation of findings [45, 51].

At the intermediate stage, descriptive results from quantitative data were used to select participants for the qualitative phase [45]. Key respondents were selected to fully represent the identified caring roles among caretakers (mothers, fathers, grandparents, foster parents, and siblings). The selection of participants also targeted to achieve proportionate representation of inclusive and special needs schools.

Integration through analysis of results was done using a convergent joint display [51]. Authors compared statistically significant quantitative results with the themes identified from qualitative results. The three authors made meta-inferences by deciding the results that converged or explained each other and prepared summary drafts. The principal investigator reviewed the drafts and resolved differences in meta-inferences [52].

**Fig. 1:**
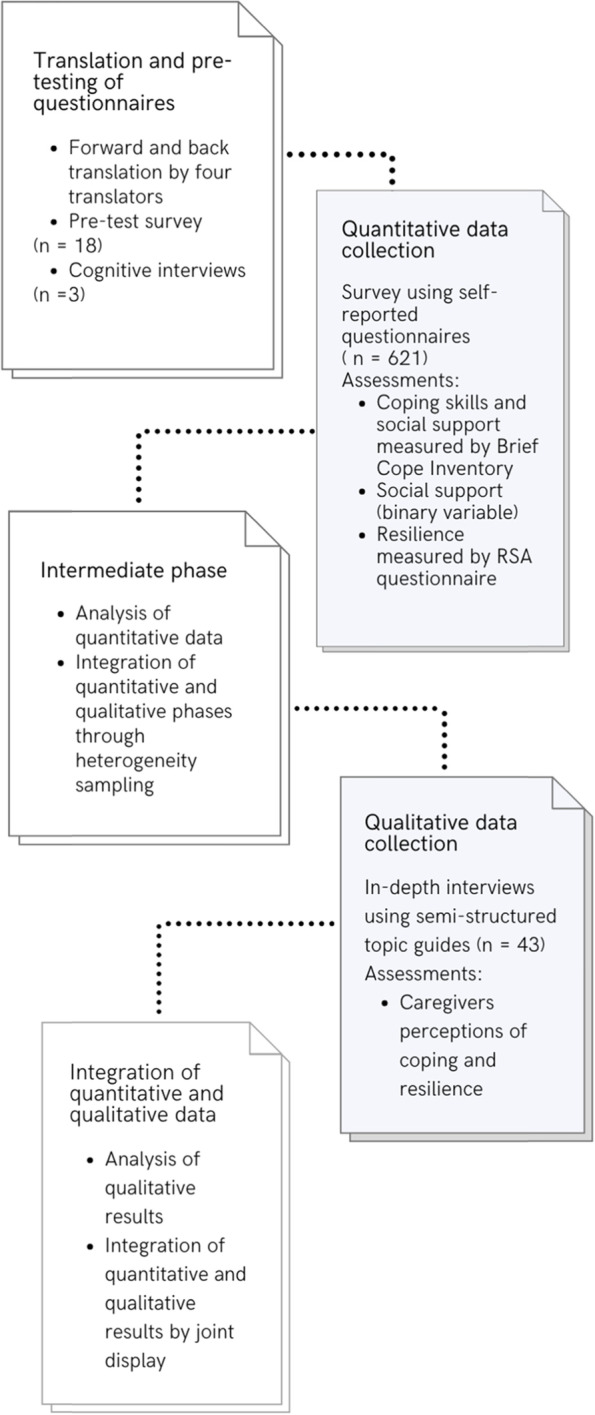
Overall study flow

The final integration point was during the interpretation of findings [53]. Meta-inferences made during analysis were used to discuss the study’s findings and clarify the underlying the factors that influenced caretakers’ perceptions of coping skills and resilience. Figure 1 shows the flow of the overall study.

### Study participants, recruitment, and data collection

#### Quantitative phase

The study used non-probability sampling to select participants for the quantitative phase [54]. The sampling frame was selected because it was the most pragmatic way of accessing caretakers of CWDs [52]. All caretakers in the participating schools were invited to join the study. A caretaker was the primary guardian of a child with mild to severe disabilities. The disabilities in children included body and functional limitations such as physical impairments, intellectual or developmental disabilities such as attention deficit hyperactivity disorder or down’s syndrome, and multiple disabilities such as cerebral palsy. The caretakers also had to be at least 18 years of age and have lived with a child with disabilities for more than six months [38]. Lastly, the caretakers had to speak either English or *Luganda* (The most common local language used in Kampala). Caretakers who did not meet the inclusion criteria and those with children above 18 years were excluded from the study. No other exclusion criteria were included in the study.

The researchers sent circulars, inviting caretakers to an information and data collection seminar. At the information seminar, caretakers who agreed to join the study were screened based on the eligibility criteria. After screening, two research assistants distributed questionnaires to participants, explained all questionnaire items and demonstrated how to fill in responses. Caretakers took 30–40 min to answer the questionnaire. Caretakers who could not read had the questions read aloud and selected responses themselves. The research assistants received a two-day training on data collection and ethical considerations before data collection.

### Qualitative phase

Caretakers were selected using a heterogeneity sampling method [55]. The selection method was based on the premise that diverse characteristics in the sample would capture caretakers’ core experiences and illuminate underlying reasons for differences in their outcomes [56]. Two research assistants used semi-structured guides to conduct in-depth interviews in *Luganda*. The interview guides had open-ended questions spanning themes on caretakers’ perceptions of coping and resilience. The first author and two research assistants transcribed the data verbatim and translated it into English for analysis.

### Questionnaires and measures

Two language experts translated all questionnaires from English to *Luganda*, and two other language experts translated them back to English for confirmation. The four translators were bilingual and had native-level command of both English and *Luganda.* The English and *Luganda* questionnaires were pre-tested in a survey with 18 caretakers. The pre-test survey assessed the length, flow, and ease of administering the questionnaire [57]. Of the 18 caretakers, three were randomly selected to examine the question-and-answer process of the questionnaire items [58]. The three caretakers participated in cognitive interviews where each respondent said out loud their cognitive process of forming judgments and selecting responses [59].

 Each participant explained their interpretation of the questions and comprehension of words used in the questionnaires. Words and phrases that were difficult for participants to understand were substituted with simpler words. The principal investigator and translators held discussions and finalized the *Luganda* and English versions of the questionnaires for the main study.

### Assessment of coping skills and social support

The study measured coping skills using the Brief Cope Inventory [60]. Brief Cope Inventory is a self-administered questionnaire with 28 items and 14 subscales. The subscales are self-distraction, active coping, denial, substance use, emotional support, instrumental support, behavioral disengagement, venting, positive reframing, planning, humor, acceptance, religion, and self-blame. The Brief Cope Inventory was preferred to alternatives such as *The Ways of Coping Questionnaire* because it includes emotional and instrumental social support as coping skills. Thus, the emotional and instrumental components of social support were measured as coping skills. The Brief Cope Inventory uses a 4-point Likert scale that indicates the extent to which a person uses different coping skills. The options are 1 (I have not been doing that at all), 2 (I have been doing this a little), 3 (I have been doing this a medium amount), and 4 (I have been doing this a lot). The minimum possible score for the whole scale is 28, and the maximum is 112. The minimum possible score for each subscale is 2, and the maximum is 8.

### Assessment of resilience

Resilience was assessed using the Brief Resilience Scale for Adults (RSA) [61]. The RSA is a 5-point Likert scale with 28 items that evaluate resilience as a sum of family and social protective factors. The most consistently reported underlying factors of the RSA are (1) perception of self, (2) planned future, (3) social competence, (4) family cohesion, (5) social resources, and (6) structured style [62, 63]. Caretakers answered 1 = not at all, 2 = a little, 3 = somewhat, 4 = quite a bit, and 5 = a lot. The least possible score of the RSA is 28 and the highest score is 140. The RSA was selected because it was the most stable scale for measuring resilience among international participants in South Africa [64].

### Measurement of covariates

**Fig. 2:**
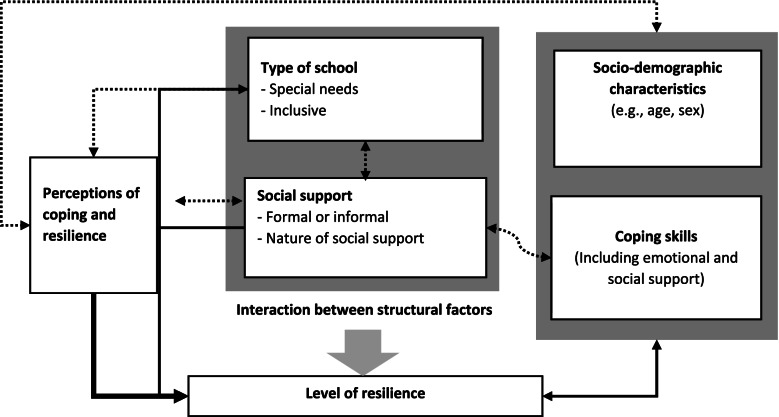
Analytic framework of the study

The study covariates were grouped into structural and socio-demographic factors. Structural factors included: the type of school (inclusive vs. special needs), nature of social support (educational materials, counseling, rehabilitation), and source of social support (school, family, and friends). Thus, the formal and informal components of social support were measured as covariates. Socio-demographic factors were caretaker characteristics (employment, age, marital status, level of education, and religious affiliation) and children’s characteristics (age, sex type, and severity of disability). The analytic framework of the study is shown in Fig. 2.

### Data analysis

#### Validation of scales

The internal consistency reliability of the scales was checked using Cronbach’s alpha [65]. The study considered values of 0.65 to 0.79 as satisfactory, and those above 0.8 and as strong [66]. The structural construct validity of the scales was examined using principal component analysis with varimax rotation [67]. Factors with eigenvalues above one were retained and used to determine the average variance extracted [68].

### Descriptive statistics

The socio-demographic characteristics of the caretakers and children were summarized using descriptive statistics.

### Assessing coping patterns among caretakers

Agglomerative hierarchical clustering was used to discern coping patterns and predict caretakers’ likelihood of using them [69]. The between-groups method was used to link the 14 coping subscales of the Brief Cope Inventory, based on an interval of Pearson’s correlation. Afterward, binary logistic regression was employed to examine the association between social support and coping skills.

### Relationships of social support, coping skills, and resilience

Independent sample t-tests and ANOVA were employed to test for statistical differences in the level of resilience among caretakers. Variables that had a p-value less than 0.02 were selected for multivariate analysis. Five hierarchical linear regression models were used to assess the relationships of social support, coping skills, and resilience. The first model included coping patterns discerned through agglomerative hierarchical cluster analysis. The second model included the 14 subscales of the Brief Cope Inventory. In the third model, structural factors were added. The fourth and fifth models considered socio-demographic characteristics of caretakers and children respectively. Multi-collinearity and the dispersion of outliers were checked in situ. Statistical Package for Social Sciences 24th version (SPSS 24) was used for all analyses, at a 5% significance level.

### Qualitative analysis

Data from in-depth interviews were analyzed using NVivo 12. Three researchers defined, labeled, and coded the data. The researchers then developed a code tree with quotations to illustrate the codes. The researchers did not assign a hierarchy to the codes or use a Kappa coefficient to test inter-item reliability. Instead, they held discussions to review each other’s codes and noted any divergence or convergence in a summary draft [70]. The researchers independently conducted a thematic analysis using the Q-sort technique and discussed the emerging themes [71]. Divergence in the themes were also included in a summary draft reviewed and resolved by the principal investigator [72, 73]. Finally, a convergent joint display was created to integrate quantitative and qualitative results [43].

## Results

### Psychometric properties of the scales

The Brief Cope Inventory showed satisfactory internal consistency reliability (Cronbach’s alpha = 0.71). The inter-item correlations of the coping subscales ranged from 0.54 to 0.91. The RSA showed strong internal consistency reliability (Cronbach’s alpha = 0.88). A complete description of the psychometric properties of the scales is provided in Supplementary Table 1.

### Quantitative results

All schools (15), with a potential population of 1000 caretakers accepted the invitation to participate in the study. Of all, eight were inclusive schools, and seven were special needs schools. A total of 670 caretakers (67%) responded to the study invitation. One school (49 caretakers) dropped out of the study, citing administrative inconveniences. Thus, the final analysis included 621 caretakers from 14 schools.

### Socio-demographic characteristics of caretakers and children

The socio-demographic characteristics of the caretakers and children are summarized in Table 1. The mean age of caretakers was 35 (10.7 SD) years, and the majority were female caretakers (81.0%). More than half (56.8%) of the caretakers were employed, with about a quarter (25.9%) having completed secondary school. The children had a mean age of about 10 (5.42 SD) years. The youngest child was three years and the oldest 18 years. Almost half (51%) were female children. The children had mild to severe physical, intellectual, or multiple disabilities. Of all, 55% had physical disabilities, and approximately 46% had mild to moderate impairments. Supplementary Table 2 shows the types of disabilities among the children.

**Table 1:** Socio-demographic characteristics of caretakers and children, by type of school (*n* = 621)

Variable	Category	Special needs school	Inclusive schools	Frequency n (%)
1) **Caretakers’ characteristics**				
**Age (yrs.)**	[mean age; SD = 35.4; 10.7]	[mean age; SD = 35.0; 10.5]	[mean age; SD = 41.2; 10.0]	
	18–25	72	4	76 (12.2)
	26–35	202	49	251 (40.4)
	36–44	111	63	174 (28.0)
	45 and above	68	52	120 (19.3)
		**453**	**168**	**621**
**Gender**	Male	90	28	118 (19.0.)
	Female	363	140	503 (81.0)
		**453**	**168**	**621**
**Employment**	Yes	235	118	353 (56.8)
	No	218	50	268 (43.2)
		**453**	**168**	**621**
**Education level**	Incomplete primary	75	62	137 (22.1)
	Complete primary	56	20	76 (12.2)
	Incomplete secondary	133	28	161 (25.9)
	Complete secondary	50	16	66 (10.6)
	Higher than secondary	25	9	34 (5.5)
	Complete university	66	23	89 (14.3)
	Vocational school	47	10	57 (9.2)
2) **Children’s characteristics**				
**Age (yrs.)**	[mean age; SD = 9.75; 5.42]	[mean age; SD = 8.51; 4.60]	[ mean; SD = 11.15; 3.55]	
	3–6	158	7	175 (28.2)
	7–13	223	104	327 (52.7)
	14 − 8	72	47	119 (19.1)
		453	168	621
**Gender**				
	Female	239	65	304 (49.0)
	Male	214	103	317 (51.0)
		**453**	**168**	**621**
**Type of disability**				
	Physical	254	88	342 (55.1)
	Intellectual	129	60	189 (30.4)
	Multiple	70	20	90 (14.5)
		**453**	**168**	**621**
**Severity**				
	Mild	48	18	66 (10.6)
	Moderate	158	60	218 (35.1)
	Severe	144	57	201 (32.4)
	Very severe	103	33	136 (21.9)

*SD* standard deviation

### Coping patterns among the caretakers

**Fig. 3:**
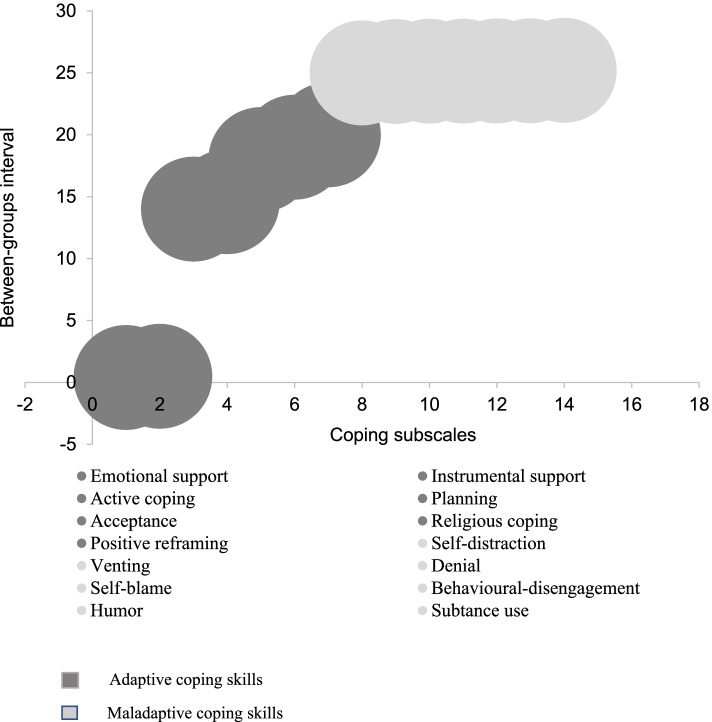
Patterns of coping based on agglomerative hierarchical cluster analysis

Figure 3 shows the coping patterns (adaptive and maladaptive coping) discerned through agglomerative hierarchical cluster analysis. Two patterns: *adaptive* (38%) and *maladaptive* (62%) coping skills were identified. Adaptive coping skills combined subscales of emotional and instrumental support, active coping, planning, acceptance, religion, and positive reframing. Of the seven subscales, emotional and instrumental support had the strongest correlation (*r* = 0.5). Maladaptive coping skills combined subscales of self-distraction, venting, denial, self-blame, behavioral disengagement, humor, and substance. Of the seven subscales, self-distraction and venting had the strongest correlation (*r* = 0.3).

### Factors associated with coping patterns among caretakers

Table 2 shows the multivariate binary logistic regression of the factors associated with coping patterns among caretakers (PCA = 68.4%). The adjusted odds ratio (AOR) is reported. Caretakers of children in special needs schools were more likely to use maladaptive coping skills than those of children in inclusive schools (AOR = 1.53; 95% CI = 1.02 to 2.30; *p* = 0.035). Caretakers who received any form of social support (formal or informal) were more likely to use adaptive coping skills than those who did not (Formal social support from school: AOR = 1.78; 95% CI = 1.20 to 2.57; *p* = 0.013, informal social support from family: AOR = 2.02; 95% CI = 1.15 to 3.54; *p* = 0.032). Caretakers of children aged between 3 and 6 years were less likely to use adaptive coping skills than those of older children (AOR = 0.38; 95% CI = 0.21 to 0.66; *p* = 0.013).

**Table 2:** Multivariate binary logistic regression of the factors associated with coping patterns among caretakers (*n* = 621)

	Variable	AOR Ex (B)	95% CI		***P***-value
			Lower	Upper	
**Child characteristics**	Child’s age group (3-6 years; maladaptive coping skills)	0.38	0.21	0.66	**0.013**
	Child’s sex (Boy)	0.91	0.63	1.31	0.588
	Type of disability (Physical disabilities)	0.77	0.45	1.32	0.200
	Severity of disability (mild)	0.89	0.74	1.07	0.222
**Structural characteristics**	Type of school (Ref: inclusive school)	1.53	1.02	2.30	**0.035**
	Formal social support from school (Ref: no)	1.78	1.20	2.57	**0.013**
	Informal social support from family (Ref: no)	2.02	1.15	3.54	**0.032**
**Caretaker characteristics**	Caretaker’s education level (Primary school)	1.05	1.00	1.14	**0.008**
	Employment status (Ref: unemployed)	1.09	0.74	1.59	0.927
	Caretaker's age (Ref: 18-25yrs.)	1.28	0.63	2.58	0.250

*Ref* reference group, *AOR* Adjusted odds ratio, *CI* confidence interval, *Ref* reference group

#### Relationships of social support, coping skills, and resilience among caretakers

Table 3 shows the fifth model of multivariate linear regression of the associations among social support, coping skills, and resilience (Model 5: R-square = 24.4%; *p* < 0.001). Caretakers who received formal social support from school had higher resilience scores than those who did not (B = 2.33; 95% CI = 0.24 to 4.40; *p* = 0.031). Caretakers who received informal social support from family showed no significant variation in their resilience scores (B = 2.49; 95% CI = -0.48 to 5.24; *p* = 0.091). Also, caretakers of children in inclusive schools had lower resilience scores than those of children in special needs schools (B = -3.38; 95% CI= -5.73 to -1.04; *p* = 0.005). Caretakers who used active coping, emotional support, instrumental support, and religious coping skills had significantly higher resilience scores than those who used other adaptive coping skills (B = 1.24; 95% CI = 0.31 to 2.17; *p* = 0.014, B = 0.99; 95% CI = 0.21 to 1.78; *p* = 0.012, B = 1.36; 95% CI = 0.66 to 2.60; *p* < 0.001, B = 1.13; 95% CI= -0.43 to 1.84; *p* < 0.001). Caretakers who used behavioral disengagement, self-blame, and self-distraction had significantly lower resilience scores than those who used other maladaptive coping skills (B = -0.70; 95% CI = -1.28 to -0.12; *p* = 0.024, B = -0.66; 95% CI = -1.21 to -0.11; *p* = 0.003, B = -0.49; 95% CI = -1.04 to 0.06, *p* = 0.025). Lastly, caretakers who had older children also had higher resilience scores than those with younger children (B = 1.87; 95% CI = 0.31 to 3.43; *p* = 0.021).

**Table 3:** Model 5 of hierarchical linear regression of factors associated with resilience among caretakers (*n* = 621)

	Variable	B	95% CI		SE_B	***P***-value
			Lower	Upper		
**Coping skills**	Coping cluster (Ref: Maladaptive coping skills)	2.58	-1.02	6.18	1.83	0.225
	Active coping	1.24	0.30	2.17	0.48	**0.014**
	Denial	0.15	-0.37	0.67	0.27	0.521
	Substance use	0.66	-0.55	1.88	0.62	0.489
	Emotional support	0.99	0.21	1.78	0.40	**0.012**
	Instrumental support	1.36	0.66	2.06	0.36	**<0.001**
	Behavioral disengagement	-0.70	-1.28	-0.12	0.30	**0.024**
	Venting	0.66	0.01	1.30	0.33	0.164
	Positive reframing	0.40	-0.26	1.05	0.33	0.151
	Planning	0.64	-0.17	1.45	0.41	0.117
	Humor	-0.15	-0.85	0.56	0.36	0.259
	Acceptance	0.67	-0.08	1.43	0.38	0.058
	Religious coping	1.13	-0.43	1.84	0.36	**<0.001**
	Self-blame	-0.66	-1.21	-0.11	0.28	**0.003**
	Self-distraction	-0.49	-1.04	0.06	0.28	**0.025**
**Structural factors**	Type of school (Ref: Special needs schools)	-3.38	-5.73	-1.04	1.10	**0.005**
	Formal social support from school	2.33	0.27	4.40	1.05	**0.031**
	Informal social support from family	2.49	-0.48	5.24	1.46	0.091
**Socio-demographic factors**	Caretaker’s education level (Ref: complete primary)	0.62	0.08	1.15	0.27	**0.017**
	Employment status (Ref: no employment)	3.53	5.57	1.48	1.04	<0.001
	Caretaker's age (Ref: 18-25yrs.)	0.20	-0.93	1.33	0.57	0.023
	Child's age * (Ref: 3-6 yrs.)	1.87	0.31	3.43	0.54	0.021

*: *p*-value < 0.05; **: *p*-value < 0.001; Ref: reference group, *B* unstandardized co-efficient, *CI *confidence interval, *SE_B *standard

Model 5: R-square = 24.4%; *p* < 0.001

## Perceptions of coping and resilience among caretakers

Table 4 shows the characteristics of participants in the qualitative study. A total of 43 respondents were selected. Of them, 25 caretakers were from inclusive schools, and 18 were from special needs schools. Among the key respondents, ten were school principals, five were class teachers, two were occupational therapists, and six were dormitory staff. Among home caretakers, 20 interviews were conducted. A total of ten home caretakers were parents of the children (Five fathers and five mothers), and ten were family relatives (grandmothers and close relatives).

**Table 4:** Age and gender profiles of participants in the in-depth interviews (*n* = 43)

Participant (n)	Mean age (yrs.) [range]	Average years of experience	Gender (%)
School principal (*n* = 10)	49 [36-55]	9	Male (100)
Class teacher (*n* = 5)	36 [19-50]	10	Male (63)
Occupational therapist (*n* = 2)	33 [27; 40]	9.5	Male (100)
Dormitory caretakers (*n* = 6)	35 [22-48]	11	Female (90)
Home caretakers (*n* = 20)	34 [18-55]	NA	Female (95)

The qualitative methods and reporting of results followed the Consolidated Criteria for Reporting Qualitative Studies (COREQ) guidelines [44] and the standard for reporting Qualitative Research (SRQR) [45]. A complete COREQ checklist is included as supplementary Tables 3, and a STROBE statement is also provided as supplementary Table 4.

## Caretakers’ perception of coping

Two themes on coping were identified: *disengaged* and *engaged* coping. Quotes from respondents are cited to illustrate the thematic results. Pseudonyms were used instead of respondents’ real names.

## Disengaged coping

Caretakers who used *disengaged* coping had difficulty relating to their children. Respondents explained that disengaged caretakers commonly denied and abandoned their children. One dormitory staff elucidated by saying:

“*Some parents deny their children and say this child is not mine, they are adopted, yet you can see the resemblance; they consider these children as unimportant; these children always come last compared to others*.“ (Dormitory staff: female, 48yrs.)

Two school principals also echoed this theme and said:

“*Male caretakers, especially fathers, deny their children saying in my lineage there are no deaf children; they do not even look at the children with love. For deaf children, facial expressions are important because that is how they judge emotions.“*

(School principal: male, 52yrs.)

*“Some (caretakers) abandon their children here for years; they even deny the children saying they are adopted, yet one can see a striking resemblance.“*

(School principal: male, 48yrs.)

Disengaged caretakers were also apathetic. Key respondents explained that apathetic caretakers rarely visited schools or sought support. The caretakers often had children their dropping out of school. An occupational therapist and a dormitory staff explained that:

“*Some caretakers are unmotivated and want everything done for them; they want things on a silver platter; even when I tell them to do something, they are not co-operative; they want me to do everything and expect miracles at the end of the school term.“*

(Occupational therapist: male, 27yrs.)

*“Some caretakers only do the bare minimum for these children;“ for example, Nammuddu’s grandmother brings her back to school with USD 0.1 to use for the whole term. She says that is all she can afford, but I know it is not true; there are times Nammuddu stays at school for months after the school term ends; the grandmother says she cannot afford to pick her up.”*

(Dormitory staff: female, 48 yrs.)

### Engaged coping

Caretakers who used *engaged coping* were identified through their connection to their children. The caretakers tended to be proactive and had accepted their children’s disabilities. They often sought support from school staff and health practitioners. Key respondents spotlighted this theme by saying:

* “Some caretakers accept and learn to accommodate their children; they even learn to love them; We see them during school activities.“*

(Class teacher: male, 40yrs.)

* “There are those who seek more knowledge and skills to take care of their children; they partner with other people concerned with children with disabilities.“*

(School principal: male, 55yrs.)

## Perception of resilience among caretakers

Three themes on the perception of resilience were discerned from the interviews with caretakers. The themes identified were *social support, socio-economic status*, and *spirituality*.

## Social support

Caretakers who received formal or informal social support felt hopeful and encouraged to care of their children. One mother expressed gratitude for the empathy that she received from friends and other caretakers:

“*Support from friends makes me strong; I used to go to a private hospital and was alone, but when I started bringing the child to school, I saw others with children like mine and was welcomed by other caretakers; I got strength knowing I am not alone; in the beginning, I was struggling, but now I am okay.“*

(Mother, 27yrs.)

Another mother explained that receiving support from professional health practitioners in school is vital for her child’s wellbeing:

*“I often bring Martina here to see a physical therapist. That has been very helpful, and it refreshes my commitment to take care of her.“*

(Mother, 32yrs.)

Stills, one mother mentioned that the school’s sign language classes had equipped her to communicate with and understand her child:

“*When I come here* (school) *for sign language classes, I learn how to communicate with my child, and it motivates me; the school staff also give me advice, and it consoles me.*“

(Mother,45yrs.)

Another mother mentioned that being in an environment with children like her own enables her to reframe her child’s abilities in a disability sensitive framework.

*“I get strengthened seeing that my child is faring better than some other children in this school.“*

(Mother, 18yrs.)

However, some caretakers reported receiving criticism from family members, amounting to the perception that formal social support was a safer mode of care than informal social support. For example, one mother explained that the school staff encouraged her to express her true feelings and struggles, but family members sometimes criticized her. The mother lamented that:

*“I once told my sister that I felt exhausted due to the constant dependence of my child; she told me never to mention such things again because it is inhumane.“*

(Mother, 32yrs.)

A father also mentioned learning adaptive coping skills that enable him to avoid confiding in family and friends.

“*Here they teach us how to manage our stress. It helps me deal with my emotions so that I do not have to tell family members my problems.”* (Father, 42 yrs.)

## Socio-economic status

Caretakers who had good socio-economic status could deal with the challenges they faced and bounced back readily. Caretakers elucidated this theme through lamentations on their financial constraints.

*“Sometimes, I do not make any money, so I cannot bring her* (child) *to school or buy enough food for the family.“*

(Grandmother, 60yrs.)

Another key respondent re-enforced the theme by saying:

“*Caretakers from well-off families are doing well. They can even hire private therapists and buy expensive medicines that others cannot afford; Financial stability enables them to seek healthcare services and buy assistive devices for their children.“*

(Dormitory staff: male 40yrs.)

Respondents also affirmed the relevance of caretakers’ education level:

* “Highly educated caretakers can appreciate the value of bringing a child to school, so they always return them and do everything they can to support their children; less educated caretakers tend to have absent children or take them out of school altogether.“*

*(*Class teacher: male, 49yrs.)

*“Educated caretakers are concerned about their children; they are financially stable and have a positive attitude towards them.“*

(Occupational therapist: male, 40yrs.)

## Spirituality

 Caretakers who believed in a higher power reported that they had peace and had learned to love and accept their children. The caretakers could positively reframe their children’s disability and gain psychological tranquility:

*“I pray a lot, so I know we will be okay; it gives me peace with the way my child is. God gave them to me that way-He alone knows why.“*

(Mother, 35yrs.)

*“Many people have favored me because of this child, and he has been a blessing to us.“*

(Mother, 25yrs.)

## Integrated results

The quantitative and qualitative results were integrated in a convergent joint display as shown in Table 5.

**Table 5:** A convergent joint display of quantitative and qualitative results

Quantitative results		Qualitative results	Meta-inference
**Variable**	**B**	**Quote**	
1) **Social support**			
Instrumental support	1.36 **	“Caretakers who get support from institutions fare better “(Class teacher, female, 37) “I often take Martina to see a therapist. That has been very helpful.” (Mother, 32)	**Convergence**: Social support is impactful for coping and building resilience
Emotional support	0.99 *	“Support from friends makes me strong.“ (Mother, 27)	**Convergence**: Emotional support is relevant for relieving psychological distress
Informal social support	2.49	*“I once told my sister that I felt exhausted due to the constant dependence of my child; she told me never to mention such things again because it is inhumane.“* (Mother, 27)	**Expansion**: Informal social support does not preclude the possibility of criticism
2) **Socio-demographic** f**actors**			
Education level	0.62 *	“Educated caretakers appreciate services like education or physiotherapy.“ (Occupational therapist, 27)	**Convergence**: Education influences the utilization of professional services
Employment	3.53 **	“Caretakers from well-off families are doing well. They can even hire private therapists.” (Dormitory caretaker, 40)	**Convergence**: Good financial status is a robust determinant of resilience
3) **Coping skills**			
Religion	1.13 **	“I pray a lot, so I know we will be okay.“ (Mother, 35)	**Convergence**: Believing in a higher power gives caretakers hope for a promising future.

*B *unstandardized co-efficient, *: *p*-value < 0.05; **: *p*-value < 0.01

(The straight arrows represent associations analyzed through quantitative analysis and dotted arrows show the associations that were examined by qualitative analysis, the heavy straight line represents integration of quantitative and qualitative results)

## Discussion

The present study used mixed methods to examine the relationships of social support, coping skills, and resilience, and the perceptions of coping skills and resilience among caretakers of children with disabilities (CWDs) in Uganda. The quantitative results revealed three main findings:

1. 62% of the caretakers mostly used maladaptive coping skills compared to 38% who mostly used adaptive coping skills.
2. Although caretakers of children in special needs schools had a higher likelihood of using maladaptive coping skills, they had higher resilience than those of children in inclusive schools.
3. Caretakers who received social support had higher resilience than those who did not.

Qualitative results revealed the underlying factors influencing caretakers’ perceptions of coping skills and resilience. Caretakers who used engaged coping were actively involved in their children’s lives, sought formal social support from the school, and received informal social support from their peers. In turn, the caretakers had firm beliefs in their ability to overcome challenges and maintained a positive outlook on disability. Notably, some caretakers found formal social support from school to be a safer mode of care than informal social support from family.

The quantitative and qualitative results converged regarding the relationships of formal social support, coping skills, and resilience. Both results showed that caretakers who received formal social support used more adaptive coping skills and had a high level of resilience. Qualitative results further expanded the relationships of social support, coping skills, and resilience by revealing the criticism that caretakers sometimes face when receiving informal social support from family. The quantitative and qualitative results were integrated with a convergent joint display.

Caretakers of children in special needs schools were more likely to use maladaptive coping skills than those in inclusive schools. Caretakers’ predisposition to use maladaptive coping skills is likely due to the age of their children rather than the type of school. In this study, caretakers who had children aged 3–6 years had greater odds of using maladaptive coping skills than those with older children. Previous studies also support the notion that caretakers of young CWDs tend to use maladaptive coping skills [46, 47]. A possible explanation is that caretakers of young children face bouts of anxiety and are still learning to care for their children [48]. However, as the children grow, caretakers accrue knowledge from lived experience and learn to adapt to the challenges they face.

Despite being more likely to use maladaptive coping skills, caretakers of children in special needs schools had significantly higher resilience than those of children in inclusive schools. The caretakers’ resilience may be attributed to two reasons: (1) formal social support from school and (2) a sense of belonging gained through interactions with their peers.

Formal social support from schools is crucial for equipping caretakers with adaptive coping skills. In the present study, caretakers explained that the technical services provided by schools improved their children’s wellbeing and enabled them to connect with and care for their children. The caretakers identified instrumental support programs such as sign language training and at-school physiotherapy as crucial aspects of social support that benefit their children. It is plausible that the special needs schools in this study were better equipped than the inclusive schools. In that case, caretakers in special needs schools could have learned adaptive coping skills enabling them to bounce back despite having a precarious coping position. In two previous studies conducted in schools for CWDs in Uganda, special needs schools commonly hired staff skilled in special needs education, while inclusive schools mostly had mainstream staff who received no training in special needs education [49, 50].

Moreover, inclusive schools, which are often government-aided in Uganda, commonly lack assistive materials to the detriment of children and their caretakers [47]. Thus, caretakers of children in inclusive schools are forced to bear the brunt of overcoming challenges on their own. This finding indicates that school formal social support is the impetus for caretakers to use adaptive coping skills and increase their resilience. Caretakers who receive formal social support can actively engage with their children’s lives and have less perceived burden of care [52–54]. Also, in the current study, some caretakers perceived formal social support from school as a safer mode of care than informal social support from family, by which they were sometimes criticized.

Perhaps informal social support is crucial for building the wherewithal to overcome challenges but provides no practical respite. Formal social support may have been a significant predictor of caretakers’ resilience because it directly impacted their livelihood. In this study, even though informal social support was not significantly associated with caretakers’ level of resilience, caretakers greatly appreciated receiving emotional support from friends and peers. A similar finding was reported by a study conducted in South Africa [27]. In South Africa, caretakers found formal social support beneficial for providing their children’s practical needs, while informal social support provided emotional relief [33]. In the present study, caretakers sometimes received financial aid from the formal social support system at school, enabling them to save on expenses such as school fees. The caretakers could then channel the savings to acquire assistive devices or access basic living necessities, improving their resilience.

Lastly, a sense of belonging gained through peer-to-peer interactions. Caretakers of children in special needs schools were encouraged by an environment with children like their own. The caretakers compared their children to others with disabilities, allowing them to frame their expectations in a disability-sensitive framework. Moreover, when caretakers of children with similar disabilities are connected, as in many special needs schools, they can share unique coping skills specific to their children’s needs which further improves their resilience [64]. On the other hand, caretakers of children in inclusive schools may compare them to children without disabilities. Such a lopsided comparison may heighten feelings of anxiety and disillusionment, and, in turn, caretakers may report low resilience [72].

The current study gives a comprehensive understanding of the relationships of social support, coping skills, and resilience. However, several potential methodological limitations could have influenced the results. First, the study used self-reported scales. Caretakers may have given biased responses due to misunderstanding or desire to be socially acceptable [55]. Second, the severity of children’s disabilities was based on the caretaker’s subjective reports. The caretaker’s interpretations of the severity of disability may not accurately represent the child’s experience [4]. Third, the validation of questionnaires in the current study falls short of the suggestions for studies conducted in settings with no gold standards for measuring psychological outcomes [73]. The present study examined only the scales’ content validity and internal reliability. One key study recommends establishing the cultural relevance of new scales by comparing them with psychological constructs that are locally known [73]. However, since the study’s scales showed acceptable content validity and internal consistency reliability, future studies may examine the cultural relevance by comparing the scales to others that have been validated in Uganda, for example, the Cognitive Emotion Regulation Questionnaire (CERQ) [74].  

Fourth, the study used non-probability sampling, introducing a selection bias in the sample [75]. The results may not be generalized to all caretakers of CWDs in or out of Uganda. Lastly, the study’s cross-sectional nature does not allow for conclusions on the direction of the relationships of social support, coping skills, and resilience.

## Conclusions

The study provides a holistic understanding of social support, coping skills, and resilience among caretakers of children with disabilities in Uganda. The study showed that even though caretakers of children with disabilities in special needs schools are highly likely to use maladaptive coping skills, they receive formal social support, which improves their resilience. Further, caretakers perceived formal social support from schools as a safer mode of care than informal social support from family.

The study finding indicate that schools are critical entities that should be leveraged to provide formal social support to caretakers of children with disabilities. Therefore, this study recommends that enrolling children with disabilities at a younger age in special needs schools is beneficial for building the resilience of caretakers as they learn to navigate the special circumstances that pertain to having a child with disabilities. The school and peer social support system created by this arrangement empowers the caretakers with a positive mindset that’s indispensable for theirs and their children’s health. Implementation of this recommendation is bound to improve the resilience of caretakers, improving theirs and their children’s health and wellbeing.

As this study highlights the multiple facets of the relationships of social support, coping skills and resilience among caretakers of children with disabilities, future studies would benefit from using mixed methods to investigate the determinants of the health and wellbeing of caretakers of children with disabilities.

## Supplementary Information

**Additional file 1: Supplementary Table 1. **Psychometric properties of the Brief Cope Inventory and the Resilience Scale for Adults

**Additional file 2: Supplementary Table 2.** Types of disabilities among children.

**Additional file 3: Supplementary Table 3.** Consolidated criteria for reporting qualitative studies (COREQ): a 32-item checklist.

**Additional file 4: Supplementary Table 4.** STROBE checklist.

## Data Availability

The dataset generated and analyzed in this study is available on Open Science Framework [https://mfr.osf.io/render?url=https://osf.io/6cdm4/?direct%26mode=render%26action=download%26mode=render].

## Abbreviations

AOR: Adjusted odds ratio
ICF: International Classification of Functioning, Disability, and Health
COREQ: Consolidated Criteria for Reporting Qualitative Studies
CI: Confidence Interval
CWDS: Children with disabilities
SE_B: Standard Error for Unstandardized Co-efficient
SD: Standard Deviation
PCA: Principal Component Analysis
