# Supplementary material for: Broadening the scope of social support, coping skills and resilience among caretakers of children with disabilities in Uganda: a sequential explanatory mixed-methods study
Source: BMC Public Health. 2022 Apr 8;22:690. doi: 10.1186/s12889-022-13018-x (PMC8991953; doi:10.1186/s12889-022-13018-x)
Supplement: Supplementary file 1 — Additional file 1: Supplementary Table 1. Psychometric properties of the Brief Cope Inventory and the Resilience Scale for Adults [file 12889_2022_13018_MOESM1_ESM.docx]

| **Factors extracted** | **Eigen value** | **Extracted sum of squared loadings** | |
| --- | --- | --- | --- |
|  |  | **Total variance (%)** | **Cumulative variance (%)** |
| **Brief Cope Inventory [ Kaiser-Meier-Olkin Measure = 0.67; Cronbach’s alpha = 0.71]** | | | |
| 1 | 3.11 | 12.00 | 12.00 |
| 2 | 2.75 | 10.59 | 22.59 |
| 3 | 1.70 | 6.52 | 29.11 |
| 4 | 1.47 | 5.65 | 34.76 |
| 5 | 1.35 | 5.18 | 39.94 |
| 6 | 1.18 | 4.55 | 44.49 |
| 7 | 1.11 | 4.26 | 48.75 |
| 8 | 1.04 | 4.02 | 52.77 |
| **Resilience Scale for Adults (RSA) [ Kaiser-Meier-Olkin Measure = 0.88; Cronbach’s alpha = 0.88]** | | | |
| 1 | 6.40 | 22.87 | 22.87 |
| 2 | 2.09 | 7.45 | 30.32 |
| 3 | 1.70 | 6.07 | 36.39 |
| 4 | 1.26 | 4.51 | 40.90 |
| 5 | 1.22 | 4.34 | 45.24 |
| 6 | 1.14 | 4.07 | 49.31 |
| 7 | 1.04 | 3.73 | 53.04 |

**Supplementary Table 1: Psychometric properties of the Brief Cope Inventory and the Resilience Scale for Adults (n = 621**)
