# Supplementary material for: Broadening the scope of social support, coping skills and resilience among caretakers of children with disabilities in Uganda: a sequential explanatory mixed-methods study
Source: BMC Public Health. 2022 Apr 8;22:690. doi: 10.1186/s12889-022-13018-x (PMC8991953; doi:10.1186/s12889-022-13018-x)
Supplement: Supplementary file 2 — Additional file 2: Supplementary Table 2. Types of disabilities among children. [file 12889_2022_13018_MOESM2_ESM.docx]

**Supplementary Table 2: Types of disabilities among children (n = 621)**

| Type of disability | Number (n) | Percentage (%) |
| --- | --- | --- |
| Attention Deficit Hyper Disorder (ADHD) | 3 | 0.5 |
| Autism | 31 | 5.0 |
| Visual impairment | 15 | 2.4 |
| Cerebral palsy | 135 | 21.7 |
| Hard of hearing (Deaf) | 309 | 49.8 |
| Down's syndrome | 11 | 1.8 |
| Hydrocephalus | 9 | 1.4 |
| Physically handicapped | 29 | 4.7 |
| Intellectual disability | 42 | 6.8 |
| Deaf with no speech | 27 | 4.3 |
| Missing | 10 | 1.6 |
| **Total** | **621** | **98.4** |

**Categorization of disabilities**

- Physical disabilities: Deaf, Deaf with no speech, hydrocephalus, physically handicapped, visual impairment
- Intellectual disabilities: ADHD, autism, down’s syndrome, intellectual disability (also referred to as mental retardation)
- Multiple disabilities: cerebral palsy
