# Supplementary material for: Broadening the scope of social support, coping skills and resilience among caretakers of children with disabilities in Uganda: a sequential explanatory mixed-methods study
Source: BMC Public Health. 2022 Apr 8;22:690. doi: 10.1186/s12889-022-13018-x (PMC8991953; doi:10.1186/s12889-022-13018-x)
Supplement: Supplementary file 3 — Additional file 3: Supplementary Table 3. Consolidated criteria for reporting qualitative studies (COREQ): a 32-item checklist. [file 12889_2022_13018_MOESM3_ESM.docx]

**Table S3:** Consolidated criteria for reporting qualitative studies (COREQ): a 32-item checklist

**Title** Broadening the scope of social support, coping skills, and resilience among caretakers of children with disabilities in Uganda: a sequential explanatory mixed methods study

**Authors**: Mariam Namasaba, Neo Kazembe, Seera Georgina, Ali Ayub Baguwemu

| **No. Item** | **Guide questions/description** | **Response** |
| --- | --- | --- |
| **Domain 1: Research team and reflexivity** | | |
| *Personal Characteristics* | | |
| 1. Interviewer/facilitator | Which author/s conducted  the interview or focus group? | Mariam Namasaba (MN) and Nabunje Sumaya (NS)  Donna Musimenta (DM) |
| 2. Credentials | What were the researcher's  credentials? | MN: BSc Nutrition, Msc Health sciences  NS: BSc Social Sciences  DM: BSc Pedagogy |
| 3. Occupation | What was their occupation at the time of the study? | Researchers |
| 4. Gender | Was the researcher male or  female? | Female |
| 5. Experience and training | What experience or training  did the researcher have? | MN and NS have done previous mixed-methods research projects on nutrition, disabilities, and food security. DM received a two-day workshop on data collection and ethics |
| *Relationship with participants* | | |
| 6. Relationship established | Was a relationship  established before study  commencement? | MN, NS, DM had no relationships with the participants before the study. |
| 7. Participant knowledge of the interviewer | What did the participants  know about the  researcher? e.g., personal  goals, reasons for doing the  research | Participants knew about the two interviewers’ names and affiliation. |
| 8. Interviewer characteristics | What characteristics were  reported about the  interviewer/facilitator? e.g.,  bias, assumptions, reasons,  and interests in the  research topic | Participants did not know about interviewers’ characteristics except their names and affiliation. |

| **Domain 2: Study design** | | | | | | | | |
| --- | --- | --- | --- | --- | --- | --- | --- | --- |
| *Theoretical framework* | | | | | | | | |
| 9. Methodological orientation and Theory | | What methodological  orientation was stated to  underpin the study? e.g.,  grounded theory, discourse  analysis, ethnography,  phenomenology, content  analysis | | See the manuscript (Pg. 7-13). | | | | |
| *Participant selection* | | | | | | | | |
| 10. Sampling | | How were participants  selected? e.g., purposive,  convenience, consecutive,  snowball | | See the manuscript in the method section/participants. | | | | |
| 11. Method of approach | | How were participants  approached? e.g., face-to-face, telephone, mail, email | | See the manuscript Pg. 9 | | | | |
| 12. Sample size | | How many participants were in the study? | | 43 participants.  See the manuscript Pg. 14 &17 | | | | |
| 13. Non-participation | | How many people refused  to participate or dropped out? Reasons? | | All key-informants accepted to participate, and none dropped out. | | | | |
| *Setting* | | | | | | | | |
| 14. The setting of data collection | | Where was the data collected? e.g., home, clinic,  workplace | | In-depth interviews were conducted at schools of children with disabilities in the Kampala Capital City Authority. | | | | |
| 15. Presence of nonparticipants | | Was anyone else present  besides the participants  and researchers? | | No. Caretakers were met in private classrooms. | | | | |
| 16. Description of sample | | What are the important  characteristics of the sample? | | See the manuscript Pg. 14 | | | | |
| *Data collection* | | | | | | | | |
| 17. Interview guide | | Were questions, prompts,  guides provided by the  authors? Was it pilot  tested? | | Yes, the interview guided were pilot tested with 5 key-informants. | | | | |
| 18. Repeat interviews | | Were repeat interviews  carried out? If yes, how  many? | | No. | | | | |
| 19. Audio/visual recording | | Did the research use audio  or visual recording to  collect the data? | | Yes, data were audio-recorded. | | | | |
| 20. Fieldnotes | | Were field notes made  during and/or after the | | Yes, field notes were made during interviews. | | | | |
| interview or focus group? | | | | | | | |  |
| 21. Duration | What was the duration of?  the interviews or focus  group? | | | | Approximately 30-40 minutes.  See the manuscript in the method section/  data collection. | | |  |
| 22. Data saturation | | Was data saturation  discussed? | | | Yes. | | |  |
| 23. Transcripts returned | | Were transcripts returned  to participants for comment No | | | | | |  |
| **Domain 3: Analysis and findings** | | | | | | |  |  |
| *Data analysis* | | | | | | |  |  |
| 24. Number of data coders | | | How many data coders coded the data? | | | Three (MN, NK, SG). |  |  |
| 25. Description of the coding tree | | | Did authors provide a  description of the coding tree? | | | Yes. |  |  |
| 26. Derivation of themes | | | Were themes identified in  advance or derived from the data? | | | Themes were derived from the data using a grounded approach. |  |  |
| 27. Software | | | What software, if applicable, was used to manage the data? | | | NVivo 12® software was used to code and manage the data. |  |  |
| 28. Participant checking | | | Did participants provide  feedback on the findings? | | | No. |  |  |
| *Reporting* | | | | | | |  |  |
| 29. Quotations presented | | | Were participant quotations  presented to illustrate the  themes/findings? Was each  quotation identified?  e.g., participant number | | | Yes. See the manuscript Pg. 17-22. |  |  |
| 30. Data and findings  consistent | | | Was there consistency  between the data presented and the findings? | | | Yes. |  |  |
| 31. Clarity of major themes | | | Were major themes clearly  presented in the findings? | | | Yes. |  |  |
| 32. Clarity of minor themes | | | Is there a description of diverse cases or a discussion on minor themes? | | | No. |  |  |
